# Efficiency of ^124^I radioisotope production from natural and enriched tellurium dioxide using ^124^Te(p,xn)^124^I reaction

**DOI:** 10.1186/s40658-022-00471-1

**Published:** 2022-06-06

**Authors:** Paweł Bzowski, Damian Borys, Kamil Gorczewski, Agnieszka Chmura, Kinga Daszewska, Izabela Gorczewska, Anna Kastelik-Hryniewiecka, Marcin Szydło, Andrea d’Amico, Maria Sokół

**Affiliations:** 1 Department of Nuclear Medicine and Endocrine Oncology, PET Diagnostics Unit, Maria Skłodowska-Curie National Research Institute of Oncology, Gliwice Branch, Gliwice, Poland; 2 Department of Systems Biology and Engineering, Silesian University of Technology, Akademicka 16, 44-100 Gliwice, Poland; 3 Biotechnology Center, Silesian University of Technology, Krzywoustego 8, 44-100 Gliwice, Poland; 4 Radiopharmacy and Preclinical PET Imaging Unit, Maria Skłodowska-Curie National Research Institute of Oncology, Gliwice Branch, Gliwice, Poland; 5 Department of Medical Physics, Maria Skłodowska-Curie National Research Institute of Oncology, Gliwice Branch, Gliwice, Poland

**Keywords:** 124-I, Iodine, Cyclotron, Monte Carlo, Nuclear medicine, Production, Radioisotope, PET-CT

## Abstract

**Background:**

^124^I Iodine (T$_{1/2}$ = 4.18 d) is the only long-life positron emitter radioisotope of iodine that may be used for both imaging and therapy as well as for ^131^I dosimetry. Its physical characteristics permits taking advantages of the higher Positron Emission Tomography (PET) image quality, whereas the availability of new molecules to be targeted with ^124^I makes it a novel innovative radiotracer probe for a specific molecular targeting.

**Results:**

In this study Monte Carlo and SRIM/TRIM modelling was applied to predict the nuclear parameters of the ^124^I production process in a small medical cyclotron IBA 18/9 Cyclone. The simulation production yields for ^124^I and the polluting radioisotopes were  calculated for the natural and enriched ^124^TeO_2_  +  Al_2_O_3_  solid targets irradiated with 14.8 MeV protons. The proton beam was degraded energetically from 18 MeV with 0.2 mm Havar foil. The ^124^Te(p,xn)^124^I reactions were taken into account in the simulations. The optimal thickness of the target material was calculated using the SRIM/TRIM and Geant4 codes. The results of the simulations were compared with the experimental data obtained for the natural TeO_2_ +Al_2_O_3_ target. The dry distillation technique of the 124-iodine was applied.

**Conclusions:**

The experimental efficiency for the natural Te target was better than 41% with an average thick target (>0.8 mm) yield of 1.32 MBq/μAh. Joining the Monte Carlo and experimental approaches makes it possible to optimize the methodology for the ^124^I production from the expensive Te enriched targets.

## Introduction

Radiotracers used in nuclear medicine diagnostics are substrates of normal physiological pathways (activated probes) or localize to particular targets because of specific binding interactions (targeted probes) [1]. One of the most prevalent radioisotopes for metabolic imaging and treatment is ^131^I. It is produced in nuclear reactors and is usually used to diagnose and treat different thyroid diseases. However, the accelerating demands of non-standard PET necessitate development and optimization methods and applications for emerging radionuclides, especially ^124^I. ^124^I provides better thyroid diagnostics, delivers less dose to patients and reduces the risk of thyroid stunning [2], facilitating subsequent therapy. Moreover, ^124^I is an attractive radionuclide for radiolabeling of monoclonal antibodies (mAbs), potential immunoPET imaging pharmaceuticals, due to its physical properties (the decay characteristics and a half-life suitable to study the processes with slow bio-kinetics [3]), typical and routine cyclotron production protocols, and well-established methodologies for radioiodination [4]. However, the practical implementation of ^124^I production in cyclotrons requires adapting the device configuration to the chosen production methodology but is highly supported by the EANM organisation [5].

^124^I has dual energy emission: beta radiation emissions of 1532 keV (11%) and 2135 keV (11%) and gamma emissions of 511 keV (46%), 603 keV (61%), and 1691 keV (11%). The gamma constant is 2.05E-4 mSv/hr per MBq @ 1.0 meter. The physical half-time (T_1/2_) of ^124^I is 4.18 days, its biological half-time is 120–138 days, and the effective half-time equals 4 days [6]. The intake routes for ^124^I may be ingestion, inhalation, puncture, wound or skin contamination and the radiotoxicity differs if the ^124^I is ingested (2.82E-7 Sv/Bq) or inhaled (1.69E-7 Sv/Bq) [6–8].

Several routes can be used to produce ^124^I in cyclotron—the choice of the strategy depends on the availability of irradiating particles and their energy ranges at a particular facility [9, 10]. One of the first schemes has been based on ^124^Te via the ^124^Te(d,2n)^124^I reaction [11–15]. In recent years ^124^I is produced from $^{124}$TeO$_{2}$ via the reaction $^{124}$Te(p,n)$^{124}$I [16–19]. This reaction has the advantages of using cyclotrons with the proton energies lower than 14 MeV, providing high radionuclidic purity, but its yields are rather low, being between roughly 6 and 20 MBq/μAh, depending on the effective energy range and the target composition [9, 20].

In this study, Geant4 (GEometry ANd Tracking 4) Monte Carlo simulation toolkit [21–23] was utilized. Geant4 is a toolkit for simulating the passage of particles through matter, and its functionalities include tracking, geometry, physics models and hits. In this environment, we calculated the proton beam penetration within the modelled target and compared the results with those from SRIM/TRIM software by Ziegler [24]. The model proposed by Poignant et al. [25] was used to optimize the production prerequisites of ^124^I via ^124^Te(p, n)^124^I reaction and its co-produced impurities. However, the parameters of the model were modified—the GE PETtrace cyclotron geometry was changed to reflect the geometry appropriate for IBA 18/9 Cyclone Cyclotron with Solid Target capabilities. The experimental data from the ^124^I production in IBA 18/9 Cyclone cyclotron and using Nitra Solid State Target were compared with the semi-experimental results from the process modelling. The aim was to adapt the production methodology of ^124^I to small medical cyclotrons and to optimize the types and thicknesses of degradation foils and target materials. The influence of the proton beam parameters on the production yield of ^124^I was taken into account.

## Materials and methods

**Fig. 1:**
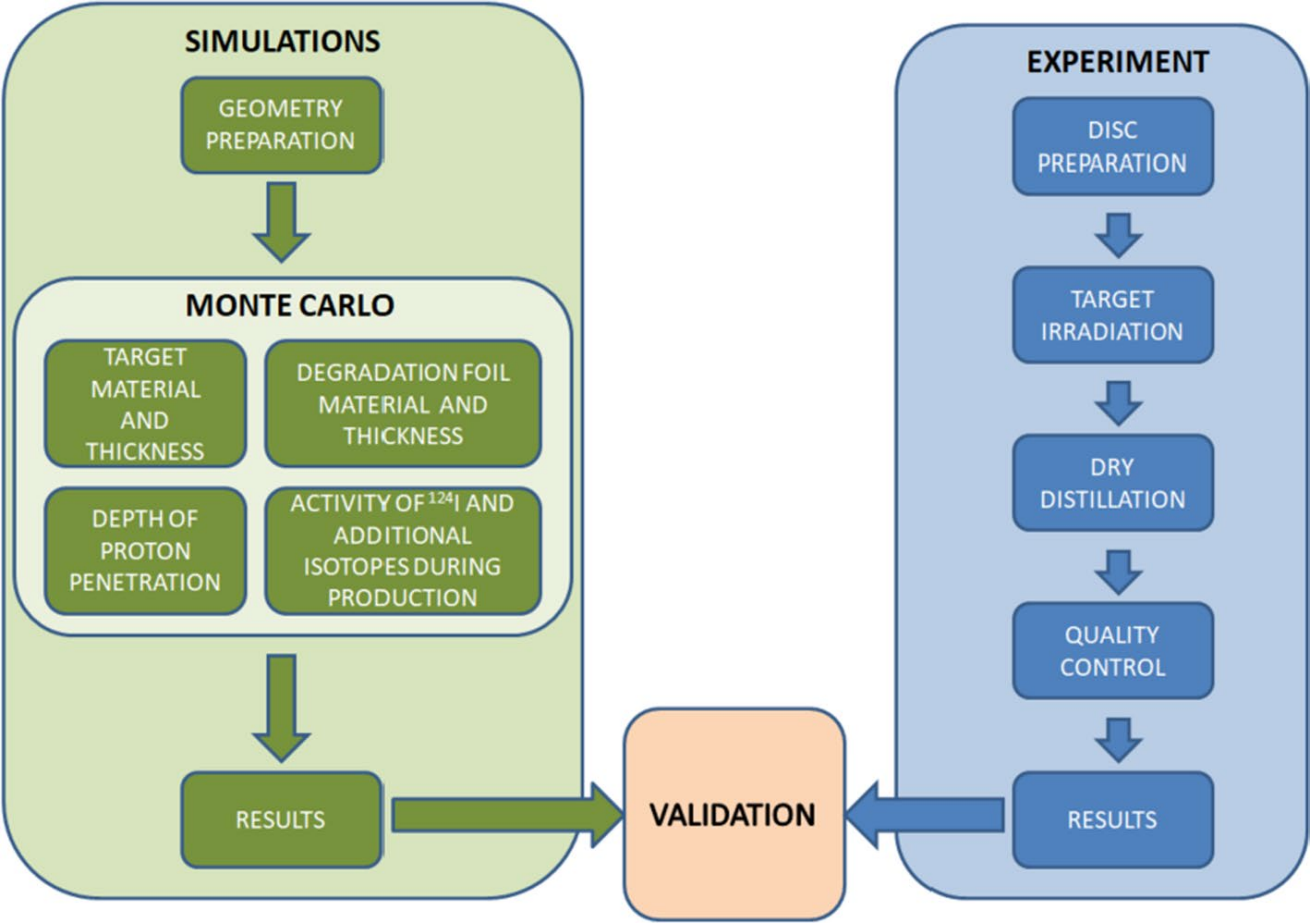
Flowchart of the Monte Carlo simulations of the $^{124}$I production and the production process experimental verification

This work consists of the semi-empirical Monte Carlo simulations and the empirical approach involving the experimental production of ^124^I from the natural Tellurium Dioxide (>99% purity, MERCK company). The workflow diagram is presented in Fig. 1.

In the experiment IBA 18/9 Cyclone cyclotron (a nominal energy of 18 MeV) installed at Maria Sklodowska-Curie National Research Institute of Oncology Gliwice Branch with a dedicated Nitra Solid COSTIS (manufactured by Elex Commerce) target for a solid material irradiation was employed.

Geant4 toolkit was applied for simulating the ^124^I production from the natural and enriched TeO_2_ targets and using various proton energy degrading foils: Havar, Molybdenum and Aluminum, as well as two proton currents: 10 μA and 15 μA. The role of the simulation parameters, such as protons energy, beam current, target material, beam energy degraders, their thicknesses and irradiation time was analyzed and optimized since these are the main sources of systematic errors and cumulative energy shifts. SRIM/TRIM (Stopping and Range of Ions in Matter), a program by Ziegler [24], was also used for validation of the production methods. This program is equipped with a graphical user interface, making the modelling much easier.

Because a sufficiently large number of particles should be simulated and tracked to obtain reasonable results, a computing cluster was used.

### Target preparation

The targetry system for production of ^124^I is limited to solid targets, usually either elemental tellurium or tellurium oxide, but the latter has better thermal characteristics as compared to the former. The natural TeO_2_ used in the experiment contains ^120^Te (0.09%), ^122^Te (2.55%), ^123^Te (0.89%), ^124^Te (4.74%), ^125^Te (7.07%), ^126^Te (18.84%), ^128^Te (31.74%), and ^130^Te (34.08%) (National Nuclear Decay Center; Brookhaven National Lab. 2009. Available online: http://www.nndc.bnl.gov/).

**Fig. 2:**
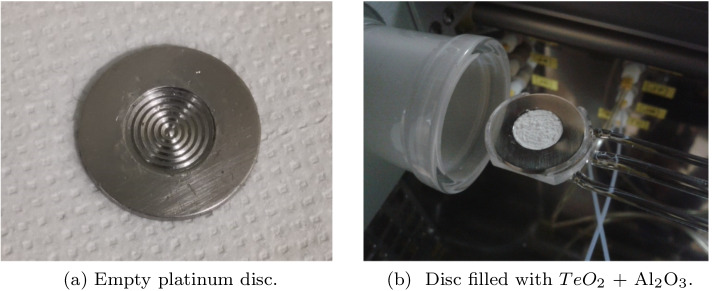
Exemplary empty target disc and target disc filled with TeO$_2$

As a target plate material, platinum was chosen, because of its high thermal conductivity (71.6 W/(m*K)) and high melting temperature (1768 °C). Such properties allow for efficient cooling during the proton beam irradiation and prevent the disc from thermal damage during TeO_2_ melting. The Pt disc has a diameter of 24 mm, a thickness of 2 mm and a 1 mm deep, circular cavity of 12 mm in the centre (Fig. 2a).

The production process starts from inserting ca. 400 mg of the ^124^TeO_2_ + Al_2_O_3_ mix (5-7wt% of aluminum oxide) into the cavity using the TERIMO module, an automatic module for separation the iodine radionuclides from the irradiated tellurium oxide targets (Fig. 2b). Al_2_O_3_ serves two purposes: to enhance the adhesion of ^124^TeO_2_ to the target disk and to produce a glassy solid matrix enhancing the materials structure [26].

**Fig. 3:**
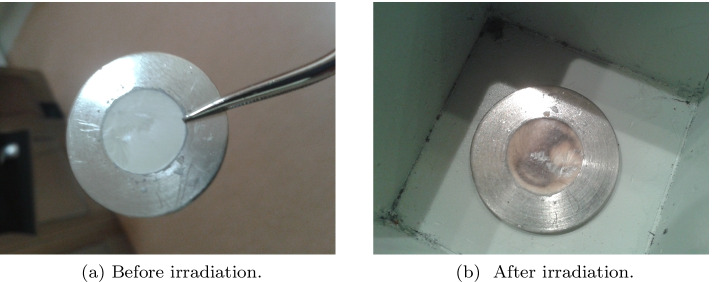
Exemplary TeO$_{2}$ target disc before (**a**) and after (**b**) irradiation

The melting of the ^124^TeO_2_ + Al_2_O_3_ mix is performed in several stages to reduce the loss of TeO_2_. First, the target is annealed at 450 °C to convert a small amount of TeO_3_ (which occasionally occurs in TeO_2_) to TeO_2_ [9]. Then, the temperature is increased up to the melting point of tellurium dioxide (733 °C) and kept for 10–20 min. After that, the mixture inside the metal disc is cooled slowly, and a glassy layer is formed, which is stuck to Pt (Fig. 3a). The tellurium dioxide glass density is 5.65 g/cm$^3$ [27, 28]. In the experiments, the melted tellurium dioxide weight within the cavity was ca. 400 mg; the filled cavity depth was ca. 0.6 mm, and the volume of the material was 0.07 cm^3^.

It is essential to optimize the amount of material on the disc. The Monte Carlo computing significantly shorten the optimization time yielding the optimal geometry and the thickness of the ^124^TeO_2_ layer.

### Target irradiation

During the bombardment, the target material was cooled with a recirculating chilled helium gas stream (gas pressure: 0.5 MPa) directed at the target, while the target holder backing was water-cooled on the front and a water flow of 16 L/min on the platinum backing. The nominal cyclotron energy of 18 MeV was moderated with a degrading foil: Havar, Molybdenum or Aluminum were applied in the simulations, whereas the experiments were performed using 0.2 mm Havar. At 14 MeV the cross-section for ^124^I is ca. 300 mb, whereas for 18 MeV it is three times lower [20, 29]. At this stage of the experiment, the proper choice of a proton beam current and a target irradiation time is crucial for production efficiency. If these parameters are too low, the amount of the produced ^124^I radionuclide is not sufficient, but if they are too high, the target could be overheated and the trapped ^124^I could leave the disc decreasing the amount of the final product. While the use of TeO$_{2}$ targets offers the benefit of re-irradiating the same target, such targets are limited to irradiation currents of typically less than 30 μA and often less than 10 μA [30]. That is why we applied two values from this rage: 10 and 15 μA. They seemed to be safe for the production process. After each irradiation, the target surface became, however, blackened (Fig. 3a, b), which occurs due to the thermal gradients caused by the interaction of protons with the target material.

### Dry distillation

A standard separation method of ^124^I from the TeO_2_ target is a dry distillation process. It consists of submitting the heated target material in a quartz tube under a gas flow, which removes the traces of TeO_2_ and traps the radioiodine while retaining the target material on the target plate.^124^I sublimes from the melted tellurium oxide (its melting point is 733 °C).

In the experiment, the process of trapping the released iodine was carried out at 750 °C. In a routine procedure, the gas with ^124^I is pumped to a solution of NaOH where it cools down and reacts to ^124^I-NaI. The flow of air transporting the iodine vapours to the NaOH solution was 100 ml/min. The flow rate of the reagents was 350 ml/min, while the transport of the products was set to 250 ml/min. The final product is a solution of ^124^I-NaI in H_2_O.

The separation was performed using the TERIMO reagent vials (B1-B5) dispensing the reagents and the cleaning solutions to the trapping vial and filled as follows:

- B1 : 1ml of NaOH
- B2 : 2ml of H_2_O
- B3 : 1ml of NaOH
- B4 : 1ml of H_2_O
- B5 : 2ml of H_2_O

A typical separation process takes approximately 90 minutes and delivers a ready-to-use product vial. The tellurium target can be used several times as each dry distillation process removes only ca. 2.5–4 mg from the disc material. Table 1 lists the target masses before and after the subsequent three bombardments as well as the corresponding mass losses for the irradiated discs 1 and 2.

**Table 1:** The target masses (with platinum disc) before and after the subsequent three bombardments as well as the corresponding mass losses for the irradiated discs 1 and 2

Disc	N$^{o}$	Mass before production [g]	Mass after production [g]	$\Delta$Mass
1	1	13.2790	13.2750	0.0040
	2	13.2750	13.2712	0.0038
	3	13.2712	13.2678	0.0034
2	1	13.0700	13.0675	0.0025
	2	13.0675	13.0642	0.0033
	3	13.0642	13.0611	0.0031

According to our observation, the method allows to get more than 75% of the total activity, while the rest goes to the WASTE vial or stays on the irradiated disc or the filters. The activity results for the product and the waste vials are presented in Table 2. A dry distillation process removes the target heterogeneities appearing after the subsequent irradiations. In this process, the target material is heated to melt (at 733$^{o}$C), whereby the surface becomes smooth, and the target can be irradiated again.

**Table 2:** Production efficiency at the end of the separation, the product (P) and waste (W) activities

Beam current [μA]	N$^{o}$	Activity P [MBq]	Activity W [MBq]	Activity P+W [MBq]	%P	%W
10	1	16.28	4.44	20.72	78.44	21.56
	2	10.36	3.70	14.06	74.12	25.88
	3	13.69	1.11	14.80	93.37	6.63
	4	17.76	0.74	18.50	95.93	4.07
	5	11.84	4.07	15.91	74.33	25.67
	6	17.39	3.33	20.72	83.25	16.75
	7	24.42	0.74	25.16	97.19	2.81
	8	22.20	1.11	23.31	94.79	5.21
	9	12.95	1.48	14.43	90.83	9.17
15	10*	52.17	4.07	56.61	92.59	7.41

The irradiation parameters: beam current 10 μA (N$^{o}$: 1–9) and 15 μA (N$^{o}$: 10 denoted with *), irradiation time of 1.5 h, Havar foil thickness of 0.2 mm

### Quality control

The last step of production is quality control (QC). First, the sterility test was performed to ensure microbiological purity. Then, after the sample ceased to radiate (about a month after a dry distillation), 0.1 ml of the product was injected into a sterile and fertile tryptic soy broth. Finally, the bottle with the broth and the sample were kept at 25$^{o}$C for four days and afterwards for another five days in 33$^{o}$C.

The gamma-ray spectroscopy was employed to acquire the radiation spectrum. The RAYTEST MUCHA multichannel analyzer with the NaI 3x3” detector was used. The 511 keV lines corresponding to the positron-electron annihilation photons and other gamma peaks from the decay were confirmed in the spectra, thus proving the suitability of sodium iodide for diagnostic use.

Finally, the radionuclide purity was tested using a Canberra-Packard gamma spectrometer equipped with high-purity germanium (HPGe) detector. The spectrum of the final product sample (of 0.5 ml volume, the spectral acquisition time of 60 minutes) was obtained two weeks after the synthesis to ensure the degradation of all radioisotopes with shorter half-lives. The spectrum was analyzed using Genie 2000 software, and the radionuclide content was determined to be below 0.1$\%$.

Similarly to other drugs for intravenous injection and according to European Pharmacopoeia [31] all radiopharmaceuticals must be formulated at and maintain an appropriate pH in order to ensure their stability, integrity and safety in medical applications. It is acceptable for the pH of the medically used radiopharmaceuticals to vary between 2 and 9 due to the blood’s high buffer capacity, and usually, the pH values of most radiopharmaceuticals are within a range of 4 to 8 [32]. In case of the radioiodine solutions, pH should be maintained at an alkaline level to avoid volatilization of iodine [33, 34]. In our experiment no pH determinations have been performed yet, because it is currently in the optimization phase of the radioisotope production conditions.

### Monte Carlo simulations

The main goals of the Geant4 simulations (Fig. 1) involved:

- optimization of the target geometry and the employed materials (as the target and the degradation foils) by selecting their types and thicknesses,
- calculation of the depth of proton penetration,
- optimization of the parameters of the $^{124}$I production process,
- characterization of the activities of ^124^I and other radioisotopes produced during the bombardment.

#### Simulation: optimization of the target and degradation foil materials and their thicknesses

The influence of the target material type (the natural vs enriched TeO_2_) and the applied degrading foil (Havar, Molybdenum or Aluminum) on the ^124^I production efficiency was analyzed. The physicochemical parameters of the simulated foil materials are collected in Table 3.

**Table 3:** The energy degrading foils characteristics for Aluminum, Molybdenum and Havar

Property	Aluminum	Molybdenum	Havar
Thermal conductivity (W m^−1^ K^−1^)	167	138	13
Melting point (°C)	582	2620	1480
Density (g cm^−3^)	2.7	10.2	8.3

In the Monte Carlo simulations, the conditions (like the target size and the type of foil material) were changing, based on the model proposed by Poignant et al. [25] to adapt the process to IBA 18/9 Cyclotron. The foils 0.05–0.5 mm thick and the targets 0.1–1 mm thick were simulated. The target thicknesses and the corresponding masses for the experimental target geometry and the material density are presented in Table 4. The simulation results were compared for two proton beam currents: 10 and 15 μA.

**Table 4:** The target thicknesses and the corresponding TeO$_{2}$ masses

Thickness [mm]	1.0	0.8	0.5	0.3	0.1
Target mass [mg]	640.9	512.7	320.5	192.3	64.1

#### Simulation: depth of proton penetration

**Fig. 4:**
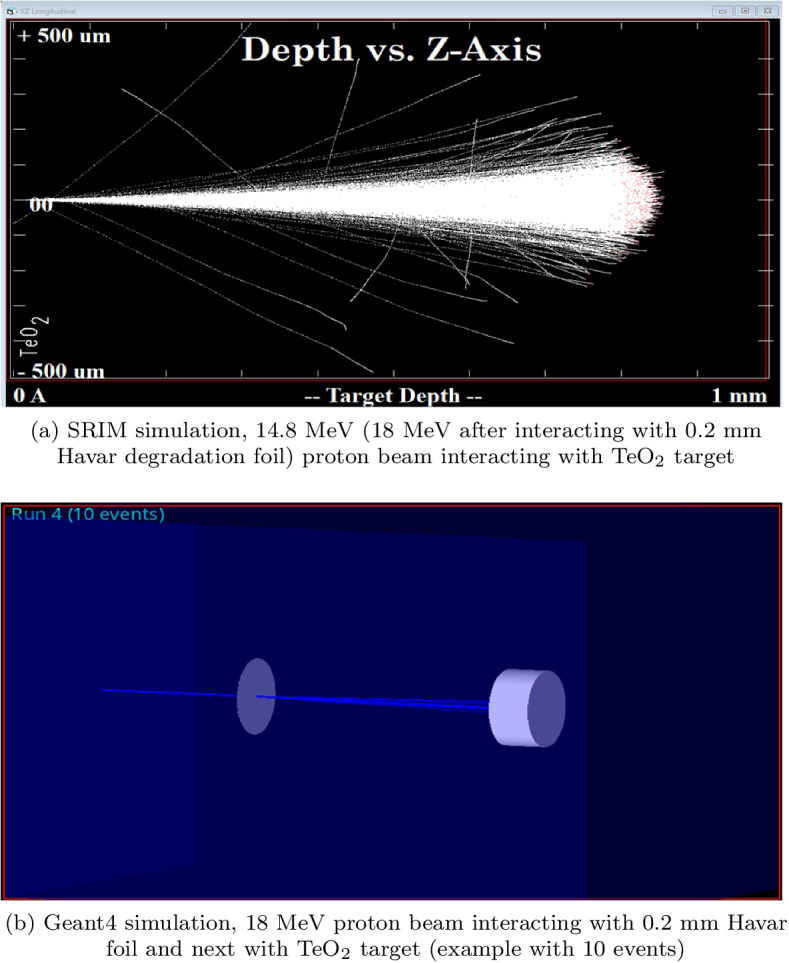
Simulated IBA 18/9 cyclotron geometries. **a** A simple SRIM/TRIM model and **b** a complex geometry of the cyclotron irradiation system created in GEANT4

SRIM/TRIM 2013 and Geant4 10.04 were used to track 14.8 MeV protons inside the bombarded target and to optimize its amount. The modelling involved more than 100 Monte Carlo simulations for 1000 iterations. Then, the stopping power and the range of incident particles in the target matter and the physical thickness of the target block were estimated. In SRIM/TRIM 2013 a simple geometry was modelled (Fig. 4a), whereas in Geant4 a complex 3D environment was created (Fig. 4b). The penetrations of protons through the target were calculated in both environments and compared. The Bethe’s formula to calculate the stopping power as a function of the proton energy E given by equation 1:

1 $$\begin{aligned} -{\frac{{\text{d}}E}{{\text{d}}x}} \ = \frac{4\pi nk^{2}_{0}z^{2}e^{4}}{mc^{2}\beta ^{2}} [ln \frac{2mc^{2}\beta ^{2}}{I(1-\beta ^{2})} - \beta ^{2} ] \end{aligned}$$

was used to fit the SRIM stopping power values. Its parameters are as follows: *k*_0_ = 8.99 x 10$^{9}$ Nm$^{2}$C$^{-2}$, (the Coulomb constant), *z*—atomic number of the heavy particle, *e*—magnitude of the electron charge, *n*—number of electrons per unit volume in the medium, *m*—electron rest mass, *c*—speed of light in vacuum, $\beta = V/c$—speed of the particle relative to c, I - mean excitation energy of the medium [35].

The ranges R of protons traveling through a medium can be calculated with the inverse of the stopping power using the formula 2:

2 $$\begin{aligned} R(E_{0})= \int _{0}^{E_{0}} \frac{{\text{d}}x}{{\text{d}}E} {\text{d}}E, \end{aligned}$$

where the integration is over the proton energy from *E*_0_, which is the energy at which the proton enters the medium, to the point where the proton has lost its energy.

#### Simulation: $^{124}$I production process

Finally, more than 100 simulations and 1000 iterations were performed in the Geant4 toolkit to simulate the ^124^I production. One thousand iterations allow for ca. 1,000,000 primaries in 15 μA and 625,000 primaries in 10 μA. The simulations were done for the TeO_2_ target containing natural Te and for that enriched in $^{124}$Te. The simulated model was created based on the GE PETtrace target system adapted to the COSTIS system of IBA 18/9 Cyclotron. The length of the target system and the target dimensions were changed. The quality of the optimized $^{124}$I production parameters were checked in the experiment performed for a natural tellurium dioxide target bombarded for 1.5 h with 14.8 MeV protons and beam currents of 10 μA and 15 μA.

### Experiment

The TeO$_{2}$ irradiation was repeated nine times using 10 μA proton beam and 34 kV RF during 1.5 h and performed only once for 15 μA proton beam and the same other parameters (unfortunately, a higher activation resulted in the cyclotron target radiation damage). The finished product was evaluated and tested through various quality checks. All activity measurements were carried out using the NUVIA ISOMED Dose Calibrator.

### PET-CT verification

The PET/CT acquisition was performed using a Biograph mCT PET/CT system manufactured by Siemens Healthcare (Erlangen, Germany) to visualize the $\beta$+ radioisotopes, like ^123^I and ^124^I. The PET/CT protocol included a standard 18F-FDG scan. A cylindrical Jaszczak phantom (Data Spectrum Corporation, Durham, NC, USA) (diameter of 21.6 cm and a volume of 6.9 L) with the micro-spheres (of the 9.5, 12.7, 15.9, 19.1, 25.4, and 31.8 mm diameters) was used for the test purposes. An iterative True-X reconstruction + TOF (the algorithm proposed by Siemens and based on the Point Spread Function (PSF) method with an additional correction) was applied. The settings of the reconstruction algorithm were as follows: 2 iterations and 21 subsets, a Gaussian filter of 3 mm, a 256 × 256 × 75 matrix (voxel spacing 3.1819 × 3.1819 × 3.00 mm) with a Time Of Flight correction. Additionally, the following $^{124}$I acquisition parameters presented elsewhere were also used for a sake of comparison:

- OSEM 4 iterations, 16 subsets, Gaussian filter 5 mm [36];
- OSEM 2 iterations, 16 subsets, Gaussian filter 6.4 mm [37];
- OP-OSEM 3 iterations, 21 subsets, Gaussian filter 5 mm [38];
- OSEM-TOF+PSF 2 iterations, 21 subsets, Gaussian filter 3 mm [39].

The settings from this study, similar to those in [39], are routinely used in the medical PET examinations at our hospital.

## Results

### Optimization of the material and the degradation foil type and thickness

**Fig. 5:**
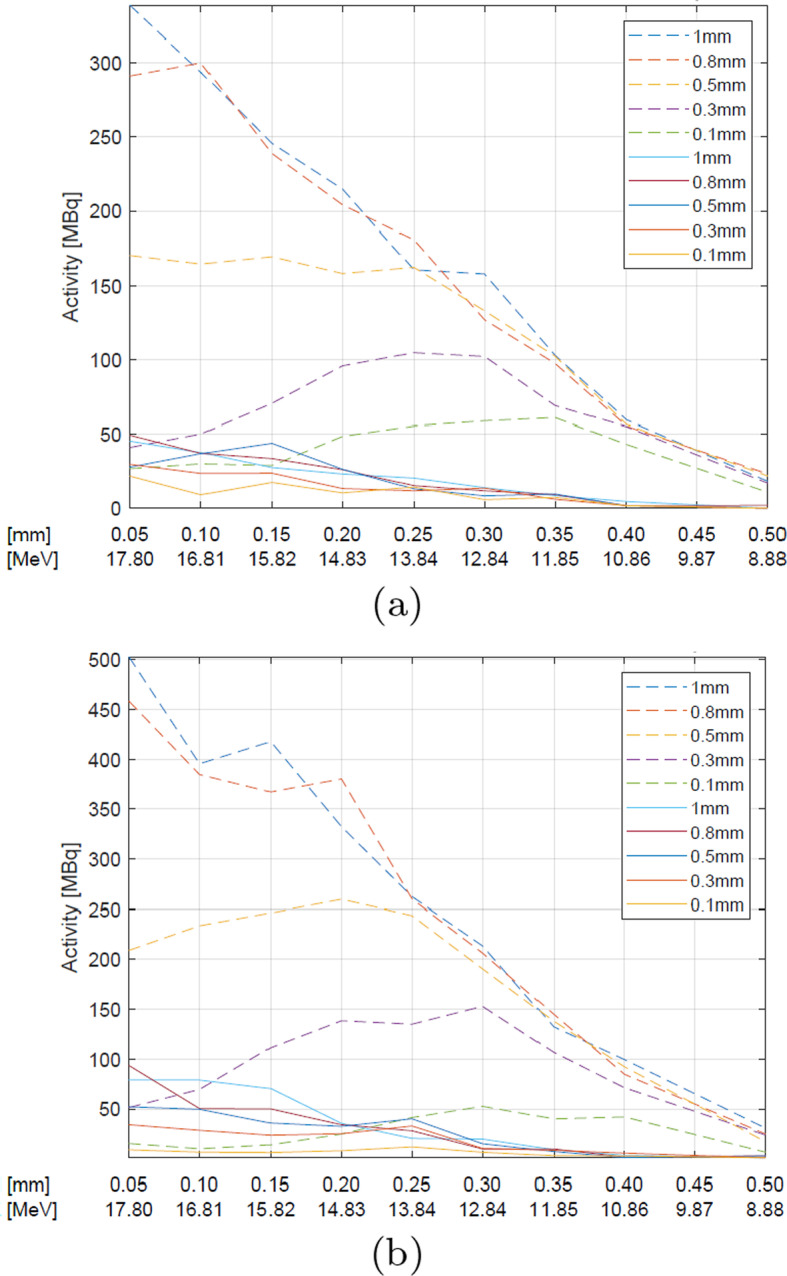
The simulated activity changes of $^{124}$I from natural (straight line) and enriched (dashed line) TeO$_{2}$ for various target thicknesses (denoted by various colors as shown in the legend) and various Havar foil thicknesses at the proton current of **a** 10 μA and **b** 15 μA

**Fig. 6:**
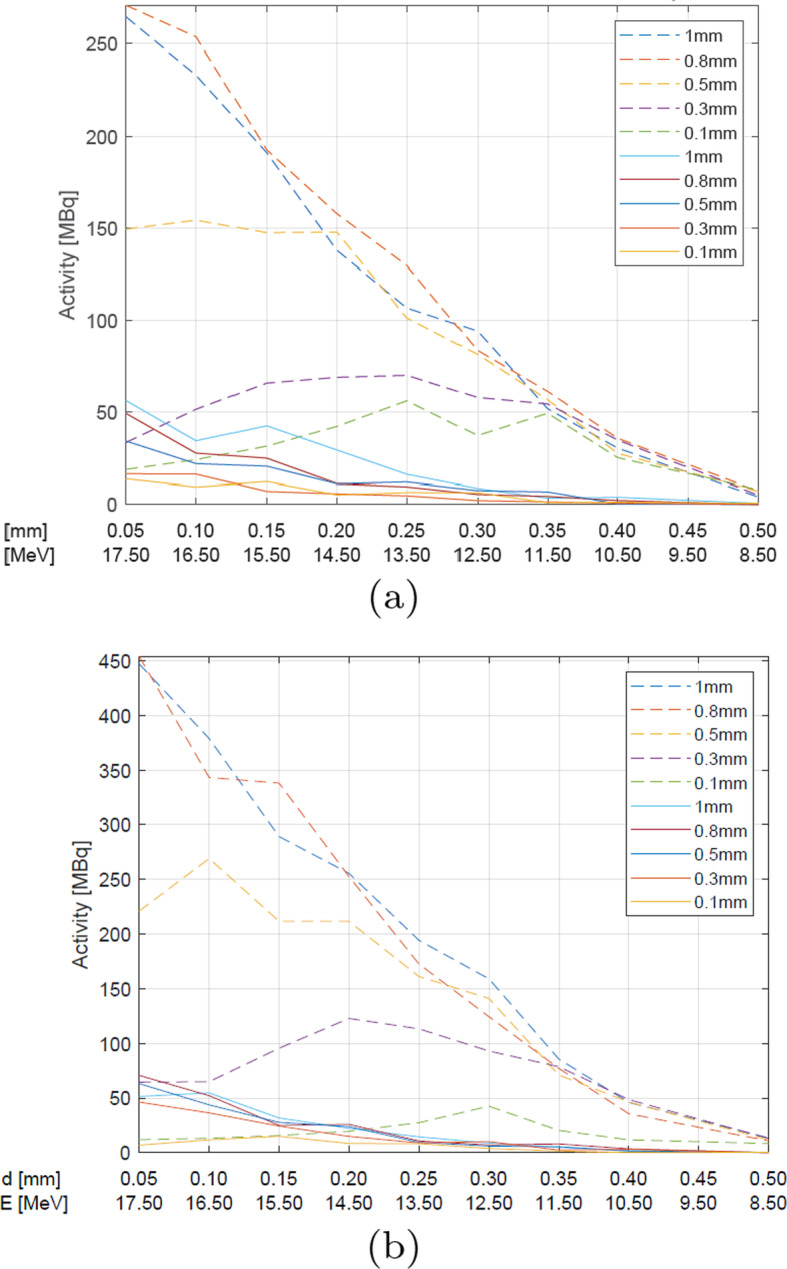
The simulated activity changes of $^{124}$I from natural (straight line) and enriched (dashed line) TeO$_{2}$ for various various target thicknesses (denoted by various colors as shown in the legend) and various Molybdenum foil thickness at the proton current of **a** 10 μA and **b** 15 μA

**Fig. 7:**
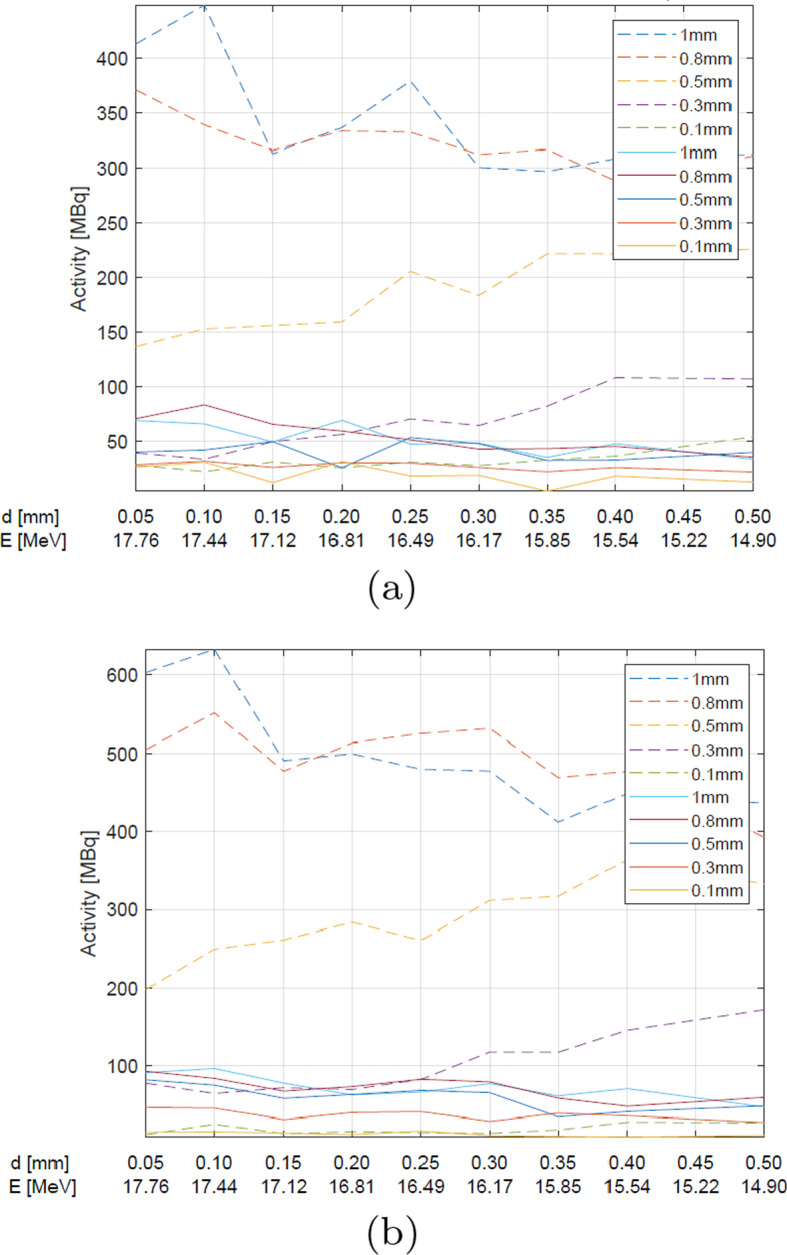
The simulated activity changes of $^{124}$I from natural (straight line) and enriched (dashed line) TeO$_{2}$ for various target thickness (denoted by various colors as shown in the legend) and various Aluminum foil thickness at the proton current of **a** 10 μA and **b** 15 μA

As reveals from Figs. 5, 6 and 7, when the target material fills the cavity of a platinum disc (the target thickness of 1 mm), the ^124^I production from natural Te requires the energies close to 18 MeV. When the target thickness is $<0.5$ mm, the maximum yield is obtained for the energies around 13 MeV, which corresponds to the maximum cross section for ^124^Te(p,n)^124^I reaction—such energies can be obtained using the degrading foil 0.2–0.3 mm thick.

In the case of the ^124^Te enriched targets, similar trends are seen, namely the energy of 17-18 MeV is optimal for the thick target (1 mm), whereas for a thinner one ($<0.5$mm) the optimum shifts to 13 MeV, like in case of natural Tellurium dioxide, however, the activity values are almost an order of magnitude higher.

At 14.8 MeV, independently of the target type and the beam current used, the amount of the produced ^124^I increases with the target thickness up to 0.5 mm, then the trend becomes reversed.

The beam current is another significant parameter affecting the ^124^I yield, as expected (Figs. 5, 6, 7). With higher current the yield should be higher, because more particles can interact with the target. As revealed from the simulations, the target thickness plays a crucial role in the ^124^I production at the energy of 14.8 MeV. For the 0.5 mm target, the maximum activity of the obtained iodine is approximately 185 MBq after 1.5 h irradiation with the proton beam of 10 μA, and 259 MBq for 15 μA. The activities up 370 MBq for 15 μA and the enriched target can be obtained for the thicker targets.

The densities of the foil materials are as follows: Molybdenum > Havar > Aluminum (Table 3). The abilities of the individual foils to slow down the proton energy with the thinnest foil layer fulfil the same relationship. In the case of the natural TeO_2_ the best results could be obtained with Aluminum foil, but only the activities of up to 74 MBq at the end of the irradiation were available (Fig. 7).

### Experimental versus simulated $^{124}$I activities

**Fig. 8:**
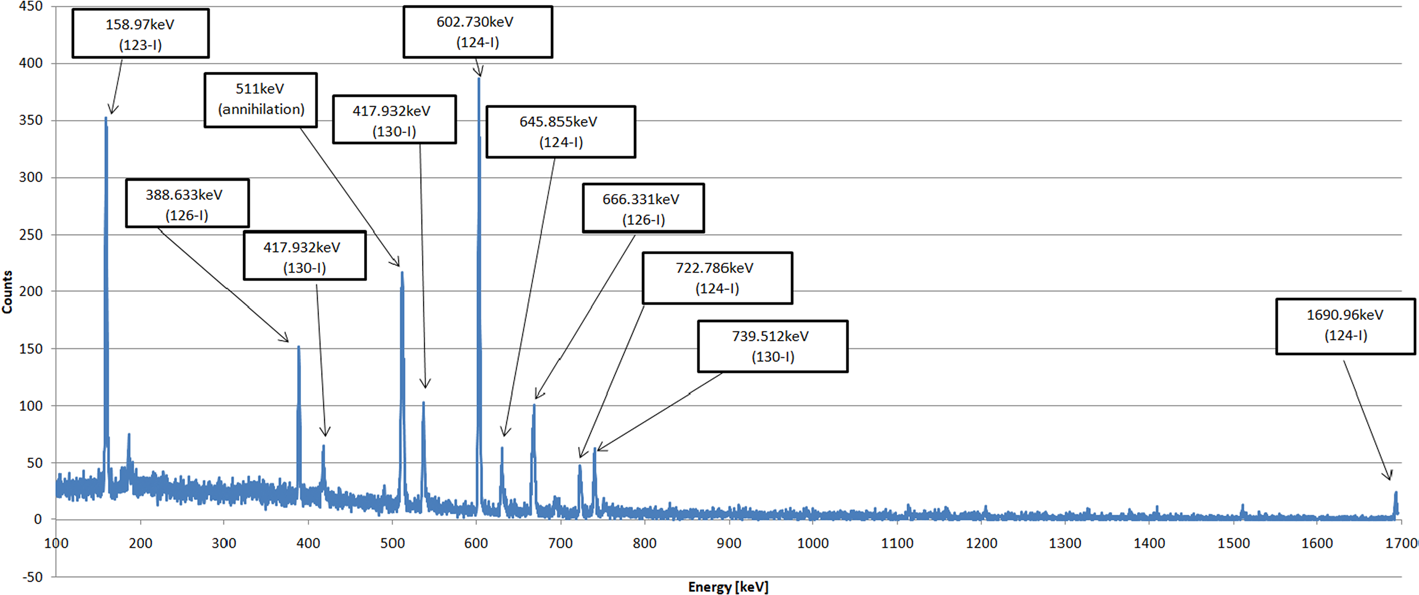
HPGe spectrum.The peaks from the $^{124}$I decay: at 603 keV, 645 keV, 722 keV and 1690 keV, and from the $^{123}$I decay (158 keV), and also $^{126}$I (388 keV and 666 keV) and $^{130}$I (417 keV and 739 keV) as well as the annihilation process (511 keV) are indicated

The test with soy broth showed no turbidity, which indicates the ^124^I NaI sample sterility. The multi-channel analyser (RAYTEST MUCHA) showed the 511 keV peaks from the annihilation processes, and the gamma spectrum revealed the peaks at 603 keV and 1690 keV from the ^124^I decay as well as the peaks from the ^123^I decay (158 keV) and the annihilation process (511 keV) (Fig. 8).

Table 5 presents the total activities after the irradiation and separation as well as the corresponding estimated values.

**Table 5:** The activities obtained in the natural TeO$_{2}$ irradiations

N$^{o}$	Time [h]	Activity EOS [MBq]	Simulated activity EOS [MBq]	Difference: experiment versus simulation [%]
1	73.88	20.72	33.30	60.71
2	118.14	13.69	24.79	81.08
3	122.77	14.43	24.42	69.23
4	96.50	18.50	28.12	52.00
5	131.36	15.91	23.68	48.83
6	72.39	20.72	34.04	64.28
7	70.44	25.16	34.41	36.76
8	70.89	23.31	34.41	47.62
9	144.87	14.43	22.20	53.85
10*	71.49	56.61	50.32	11.11

The irradiation parameters: the proton beams of 10 μA (N$^{o}$: 1-9) and of 15 μA (N$^{o}$: 10 denoted with *), irradiation time of 1.5 h, the thickness of the Havar foil: 0.2 mm. The results are obtained at the end of separation (EOS) and Time [h] is the time difference between the end of bombardment and the end of separation

The Monte Carlo simulated values were calculated for the irradiation-separation times equal exactly to the experimental values. The experimental activities at the end of separation (EOS) were compared with the activities from the Monte Carlo simulations.

For the natural ^124^TeO_2_ target the maximum estimated ^124^I activity is 34.41 MBq for irradiation parameters: 14.8 MeV, 10 μA, Havar 0.2 mm and 50.32 MBq for parameters: 14.8 MeV, 15 μA, Havar 0.2 mm. The corresponding experimental values are 25.16 and 56.61 MBq, respectively for 10 μA and 15 μA (Table 5).

### Simulation: depth of proton penetration

14.8 MeV proton penetration ranges numerically calculated for the TeO$_{2}$ target are similar in both, Geant4 and SRIM/TRIM methods and equal 812.8 ± 15.1 μm and 866.0 ± 23.7 μm, respectively.

**Fig. 9:**
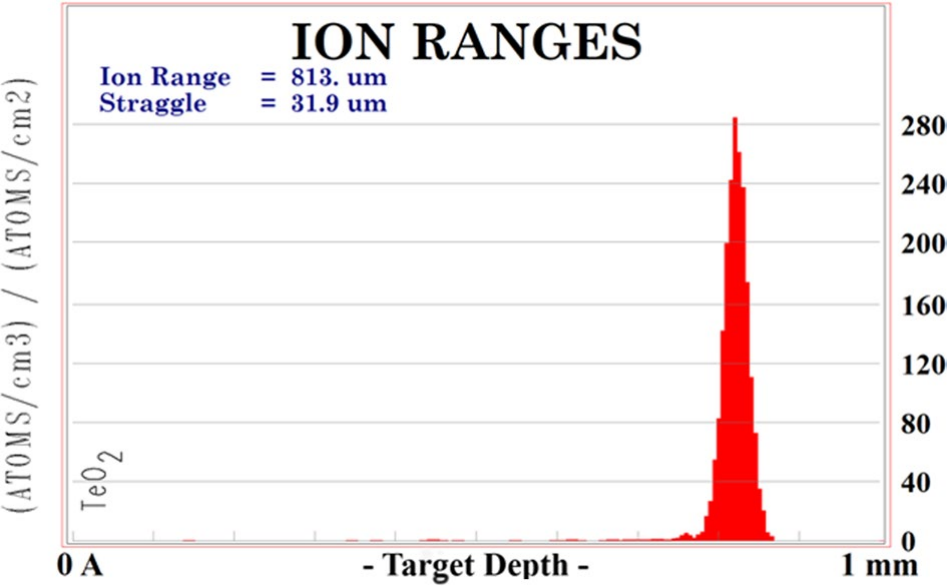
SRIM/TRIM experiment. An exemplary stopping range for protons (14.8 MeV) in Tellurium dioxide target. The peak of distribution is for 813.0 μm

The proton range depends on the capture cross-section and the material the protons interact with as they are slowed down. The energy of the bombarding beam varies with depth of penetration and the cross-section for the nuclear reaction of interest varies with bombarding energy. A large part of the protons’ energy is distributed at the end of their trajectories forming the Bragg peak, and the amount of charge determines its shape—a higher charge results in a narrower peak. As reveals from Fig. 9 and the SRIM calculations the targets of the thicknesses >0.8 mm significantly reduce the energy of the bombarding particles or completely absorb the beam; thus, such targets can be considered as thick.

**Fig. 10:**
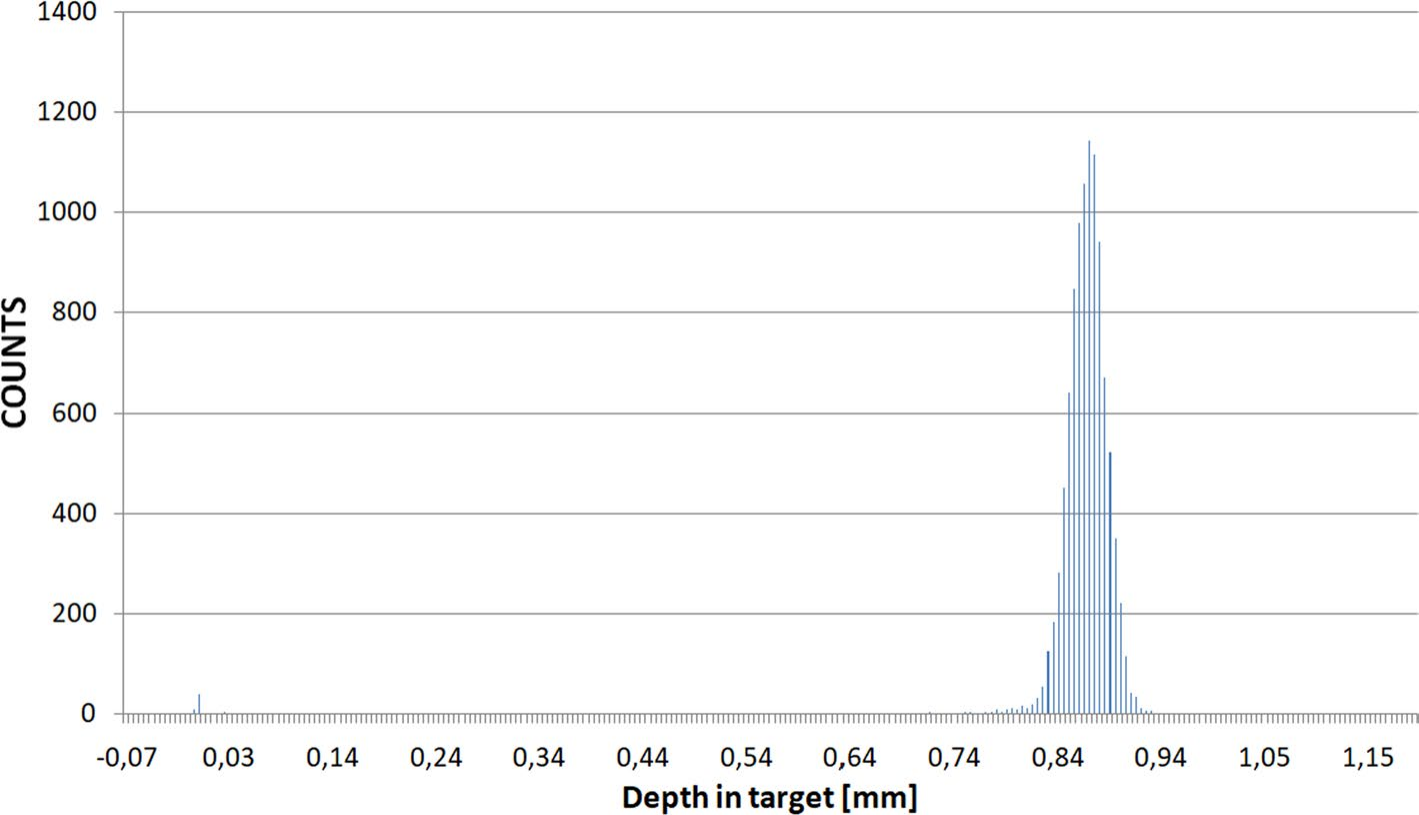
Geant4 experiment. An exemplary stopping range for protons (14.8 MeV) in Tellurium dioxide target. The peak of distribution is for 886.2 μm

Figures 9 and 10 shows the exemplary proton ranges distribution in the TeO_2_ target obtained using SRIM/TRIM 2013 and Geant4.

### Simulation: ^124^I production process

The production of ^124^I was predicted through modelling of the ^124^Te(p,n) reaction at the proton energy of 14.8 MeV, taking into account also the (p,xn) reactions when analyzing the contamination products. The simulated activities of ^124^I as well as of the impurities, like ^123^I,^125^I,^126^I and ^130^I were calculated and the results are shown in Tables 6, 7, 8 and 9.

**Table 6:** The results of the Monte Carlo simulation of the $^{124}$I production process

Radioisotope	Half Life	Decay mode	EOB [MBq]	EOB [%]	After 72 h [MBq]	After 72 h [%]
$^{6}He$	806.7 ms	$\beta$-	72.483	1.24	0	0
$^{119}I$	19.1 min	EC $\beta +$	138.010	2.36	0	0
$^{120}I$	81.6 min	EC $\beta$+	76.664	1.31	0	0
$^{122}I$	3.6 min	EC $\beta$+	717.393	12.27	0	0
$^{123}I$	13.2 h	EC $\beta$+	672.623	11.51	15.429	10.38
^124^**I**	**4.2 d**	**EC* β+***	**214.711**	**3.67**	**130.499**	**87.71**
$^{13}N$	9.9 min	EC $\beta$+	3794.831	64.92	0	0
$^{14}O$	70.6 s	EC $\beta$+	143.449	2.45	0	0
$^{119}Sb$	38.2 h	EC	3.848	0.07	1.036	0.70
$^{122}Sb$	2.7 d	$\beta$- (97.59%), EC $\beta$+ (2.41%)	1.147	0.02	0.518	0.35
$^{119}Te$	16.1 h	EC $\beta$+	8.991	0.15	0.407	0.27
$^{121}Te$	19.2 d	EC $\beta$+	0.962	0.02	0.888	0.59
Total			5845.112	100.00	148.777	100.00

The production parameters: TeO$_{2}$ **enriched** in $^{124}$Te, 18 MeV protons moderated to 14.8 MeV with 0.2 mm Havar, the proton current of 10 μA. The activity values at the end of bombardment (EOB) and the activity values after 72 hours since the end of bombardment are presented. $^{124}$I is highlighted in bold

**Table 7:** The results of the Monte Carlo simulation of the $^{124}$I production process

Radioisotope	Half Life	Decay mode	EOB [MBq]	EOB [%]	After 72 h [MBq ]	After 72 h [%]
$^{118}I$	13.7 min	EC $\beta$+	71.447	0.85	0	0
$^{119}I$	19.1 min	EC $\beta +$	69.449	0.83	0	0
$^{120}I$	81.6 min	EC $\beta$+	77.182	0.92	0	0
$^{121}I$	2.12 h	EC $\beta$+	223.904	2.67	0	0
$^{122}I$	3.6 min	EC $\beta$+	1732.821	20.69	0	0
$^{123}I$	13.2 h	EC $\beta$+	1151.958	13.75	26.455	11.46
^124^**I**	**4.2 d**	**EC β+**	**332.334**	**3.97**	**201.983**	**87.50**
$^{13}N$	9.9 min	EC $\beta$+	4972.319	59.36	0	0
$^{118}Sb$	3.6 min	EC $\beta$+	72.187	0.86	0	0
$^{119}Sb$	38.2 h	EC	1.924	0.02	0.518	0.23
$^{118}Te$	6.0 d	EC	0.518	0.01	0.370	0.16
$^{119}Te$	16.1 h	EC $\beta$+	4.514	0.05	0.185	0.09
$^{121}Te$	19.2 d	EC $\beta$+	1.480	0.02	1.332	0.57
Total			8712.02	100.00	230.843	100.00

The production parameters: TeO$_{2}$ **enriched** in $^{124}$Te, 18 MeV protons moderated to 14.8 MeV with 0.2 mm Havar, the proton current of 15 μA. The activity values at the end of bombardment (EOB) and the activity values after 72 hours since the end of bombardment are presented. $^{124}$I is highlighted in bold

**Table 8:** The results of the Monte Carlo simulation of the $^{124}$I production process

Radioisotope	HalfLife	Decay mode	EOB [MBq]	EOB[%]	After72h[MBq]	After72h[%]
$^{11}Be$	13.8 s	$\beta$-	72.483	0.87	0	0
$^{6}He$	806.7 ms	$\beta$-	72.483	0.87	0	0
$^{120}I$	81.6 min	EC $\beta$+	38.739	0.46	0	0
$^{122}I$	3.6 min	EC $\beta$+	434.972	5.19	0	0
$^{123}I$	13.2 h	EC $\beta$+	65.786	0.79	1.517	4.44
^124^**I**	**4.2 d**	**EC β+**	**23.199**	**0.28**	**14.097**	**41.47**
$^{125}I$	59.4 d	EC	3.552	0.04	3.404	10.06
$^{126}I$	12.9 d	EC $\beta$+ (52.7%), $\beta$- (47.3%)	14.319	0.17	12.173	35.84
$^{128}I$	24.9 min	$\beta$- (93.1%), EC $\beta$+ (6.9%)	3326.115	39.71	0	0
$^{130}I$	12.4 h	$\beta$-	157.916	1.89	2.775	8.19
$^{13}N$	9.9 min	EC $\beta$+	3979.609	47.51	0	0
$^{14}O$	70.6 s	EC $\beta$+	72.483	0.87	0	0
$^{15}O$	122.2 s	EC $\beta$+	72.483	0.87	0	0
$^{129}Te$	69.6 min	$\beta$-	42.92	0.51	0	0
Total			8377.059	100.00	33.966	100.00

The production parameters: **natural** TeO$_{2}$, 18 MeV protons moderated to 14.8 MeV with 0.2 mm Havar, the proton current of 10 μA. The activity values at the end of bombardment (EOB) and the activity values after 72 hours since the end of bombardment are presented. $^{124}$I is highlighted in bold

**Table 9:** The results of the Monte Carlo simulation of the $^{124}$I production process

Radioisotope	Half Life	Decay mode	EOB [MBq]	EOB [%]	After 72 h [MBq]	After 72 h [%]
$^{6}He$	806.7 ms	$\beta$-	71.817	0.86	0	0
$^{119}I$	19.1 min	EC $\beta +$	69.079	0.82	0	0
$^{120}I$	81.6 min	EC $\beta$+	38.406	0.46	0	0
$^{121}I$	2.12 h	EC $\beta$+	111.370	1.33	0	0
$^{122}I$	3.6 min	EC $\beta$+	1221.111	14.58	0	0
$^{123}I$	13.2 h	EC $\beta$+	70.596	0.84	1.628	3.23
^124^**I**	**4.2 d**	**EC β+**	**35.594**	**0.42**	**21.608**	**43.13**
$^{125}I$	59.4 d	EC	3.996	0.05	3.848	7.66
$^{126}I$	12.9 d	EC $\beta$+ (52.7%), $\beta$- (47.3%)	17.316	0.21	14.726	29.38
$^{128}I$	24.9 min	$\beta$- (93.1%), EC $\beta$+ (6.9%)	5404.738	64.52	0	0
$^{130}I$	12.4 h	$\beta$-	361.527	4.32	6.364	12.72
$^{13}N$	9.9 min	EC $\beta$+	5161.833	61.62	0	0
$^{14}O$	70.6 s	EC $\beta$+	143.671	1.71	0	0
$^{119}Sb$	38.2 h	EC	1.928	0.02	0.518	1.04
$^{122}Sb$	2.7 d	$\beta$- (97.59%), EC $\beta$+ (2.41%)	1.147	0.01	0.518	1.05
$^{119}Te$	16.1 h	EC $\beta$+	4.514	0.05	0.185	0.40
$^{121}Te$	19.2 d	EC $\beta$+	0.666	0.01	0.592	1.16
$^{127}Te$	9.4 h	$\beta$-	22.681	0.27	0.111	0.22
$^{129}Te$	66.6 min	$\beta$-	42.513	0.51	0	0
Total			12784.503	100.00	50.098	100.00

The production parameters: **natural** TeO$_{2}$, 18 MeV protons moderated to 14.8 MeV with 0.2 mm Havar, the proton current of 15 μA. The activity values at the end of bombardment (EOB) and the activity values after 72 hours since the end of bombardment are presented. $^{124}$I is highlighted in bold

As expected, when using natural tellurium dioxide, the theoretical activity of the produced ^124^I radioisotope is almost one order of magnitude smaller (23.199 MBq and 35.594 MBq for 10 μA and 15 μA, respectively) than in case of the Te enriched target (214.785 MBq and 332.334 MBq for 10 μA and 15 μA, respectively), whereas the amount of the produced impurities is much higher—60% vs 12% of the total yield (Tables 7, 8). The calculated total activities after 72 h since the bombardment are 34.003 MBq and 50.135 MBq for the proton currents of 10 μA and 15 μA, respectively (Tables 8, 9), whereas the corresponding experimental activities after 72 h are ca. 25.16 MBq and 56.61 MBq (Table 5); however, the latter value is from a single experiment.

### Measurement: ^124^I verification via PET-CT

**Fig. 11:**
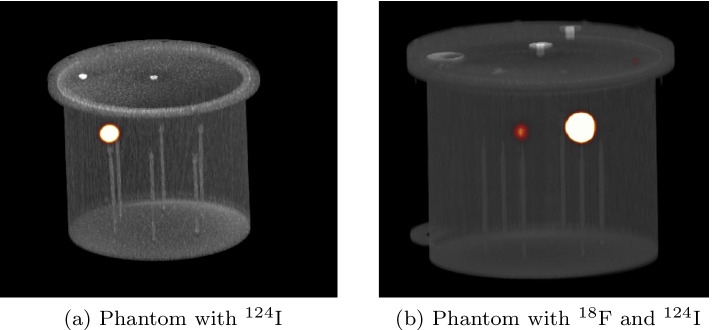
PET/CT phantom acquisition using a cylindrical Jaszczak Phantom (Data Spectrum Corporation, Durham, NC, USA), the diameter of 21.6 cm and a volume of 6.9 L, with the microspheres (9.5, 12.7, 15.9, 19.1, 25.4, and 31.8 mm diameter)

Finally, we performed the PET-CT study to capture the radioactivity emanating from $^{124}$I. Two tests were done: in the first test one sphere (diameter = 19.1 mm) was filled with the final product from the natural TeO_2_ irradiation (Fig. 11a), whereas the remaining spheres contained distilled water.

In the second test, the smaller sphere (diameter = 15.9 mm) was filled with the distilled ^124^I, whereas the larger one (diameter = 19.1 mm) with ^18^F, and the remaining ones contained distilled water (Fig. 11b). As seen in Fig. 11a, b $\beta$+ emitter positrons are present in both cases, confirming the applicability of both products in PET/CT imaging.

## Discussion

The $^{124}$I has been extensively investigated for the last several years. Although ^124^I is now well-known [40–44], its efficient extraction is still a problem, which limits its use in diagnostics and research. Also, Iodine-124 production data is vast—many alternative nuclear production pathways exist, encompassing a wide range of reactions [45, 46].

However, the clinical applications of this radioisotope are limited owing to its very high production cost and lack of widespread availability [47]. The interest in ^124^I applications is expected to grow, as it can be attached to the cell surface and used in cell labelling, opening new possibilities in the studies of human metabolism [4, 48]. Only about 23% of its disintegration is *via* positron emission of relatively high energy [6]. The other decay processes involve emissions of high-energy $\gamma$ rays, some in cascade with the positrons [7]. Gamma radiation increases the radiation dose in the patients and complicates the dose calculations; however, the $\beta$+ presence makes this radioisotope suitable for PET studies [35]. On the other hand, Auger electron emission (electron yield per decay = 8.6, [49]) gives it the capability to be named a theranostic agent [50–52]. However, a theranostic approach on Iodine-124 is not yet proved and consolidated [53]. ^124^I finds, however, its application, collectively with ^131^I, as a part of a theranostic pair [54]. Such pairs, formed of the similar and matching radionuclides, better serve diagnostic purposes by lowering radiation burden and achieving better image quality [55].

In our work the ^124^Te(p,xn) reactions, the most effective in the natural TeO_2_+Al_2_O_3_ target and in the target enriched with ^124^Te irradiated with 14.8 MeV protons, were simulated in the Monte Carlo code. The results of the simulations were compared with the experimental data obtained for the natural TeO_2_+Al_2_O_3_ target. The degradation Havar foil, optimized *via* simulations to be 0.2 mm thick, was also applied in the experiment.

As revealed from the SRIM/TRIM and Geant4 simulations, for the natural ^124^TeO_2_ targets irradiated with 14.8 MeV proton beams, the thicknesses >0.8 mm are required to markedly reduce the energy of the bombarding particles or to completely absorb the bombarding beam. A too thin layer would make protons fly through it without ^124^I production, whereas a too thick one unnecessarily increases the costs of the production, especially in the case of tellurium enriched $^{124}$TeO_2_, and the risk of the target overheating [56]. Moreover, because the dry distillation is used to separate ^124^I from the disk, the thickness of the material cannot be too great. In natural TeO_2_^124^I has a maximum cross-section at >14 MeV—so is produced very close to the surface of the target and diffuses out faster compared to ^130^I, which has its maximum cross-section at much lower energy and is, thus, produced deeper in the target [57]. Therefore, the distillation parameters should be optimized individually, which is especially important when optimizing the production from natural tellurium. As shown by Scholten et al. [19], at the incident energy of 14.8 MeV the ^123^I production from the reaction ^124^Te(p,2n)^123^I, concurrent to ^124^Te(p,n)^124^I, is slightly higher than of ^124^I [19]. The amount of the co-produced iodine ^123^I is high, but 48 h since irradiation its activity decreases below 10% of the initial value, whereas that of ^124^I drops to ca. 75%. It is due to the large difference in the half-lives of both radionuclides (Table 6).

Of interest are also the reactions (p,2n) and (p,3n) on the long-lived ^125^I (T_1/2_= 60.2 days), ^125m^Te (T_1/2_= 57.40(15) days) and the stable ^125^Te and ^126^Te nuclei. It was established that the content of the ^125^I and ^126^I impurities depends on the protons’ energy and the thickness of the target material. In the (p,n) reaction, the yield of ^126^I is somewhat smaller than that in the (d,2n) reaction; the amount of ^125^I drops markedly below that of ^126^I, and ^131^I could not be detected at all [19]. Unfortunately, due to the high natural abundances of other radioisotopes, like ^125^Te (7.07%) and ^126^Te (18.84%) in natural tellurium, the reactions like (p,2n) and (p,3n) may become of importance by increasing the amount of the radiochemical radioiodine contaminants and affecting the final yield of iodine ^124^I.

At the end of the final product separation (it contains ^124^I and the radioactive impurities), the experimental activities were 25.16 MBq and up to 56.61 MBq for 14.8 MeV protons at 10 μA and 15 μA, respectively. The Monte Carlo simulations of the reactions ^124^Te(p,xn) used to estimate the amount of the produced radioisotope based on the disk geometry and the cyclotron operating parameters yielded up to 34.003 MBq (10 μA) for the natural target, and 50.135 MBq (15 μA) for the Te enriched target. Thus, for the natural TeO_2_ there are some relative discrepancies between the simulated values and the experimental data—for the beam current of 10 μA the simulated activity is about 30% higher than the measured one, whereas, in the case of 15 μA proton beam, it is ca. 12% lower. They are presumably due to the differences in the simulated and experimental target volumes. In the simulations, the target material was assumed to fill the whole cavity, whereas in the experiments, the target material, after melting, filled up slightly less than the entire volume intended for it. Moreover, the applied proton current and the successive re-irradiations are of importance: the production yields can be enhanced by increasing the proton beam current, but the maximum beam deposited on a target is limited by the targetry, including the thermal characteristics of the target material and the cooling system to prevent a possible loss of the target material due to inhomogeneous distribution of temperature resulting in appearance of a subsequent local target burnt-up areas [58]. They disappear, however, after the dry-distillation processes. Thus, once prepared, the target can be used many times until its thickness is significantly reduced. In the experiment, the loading of about 400 mg of tellurium dioxide with aluminium oxide was applied, and the mass losses after the subsequent dry distillations were of about 2.5-4 mg. Thus, to obtain a close agreement between the simulated and experimental values, these processes should be taken into account. The experimental yield is also influenced by a beam profile and an intensity, the radiation damage effects and a chemical separation yield [57, 59]. In the case of the proton beam of 15 μA, an additional source of error is the lack of repeated measurements (due to the target disruption). Thus, the obtained activity value is presumably overestimated. Also, more repetitions would undoubtedly improve the statistical strength for the beam of 10 μA (9 re-irradiations). Furthermore, because tellurium is the radioisotopes mixture in its natural form, the radioactive pollution level is very high, as many, mainly short-lived, radioisotopes can be produced on irradiation by the (p xn) reactions and the reactions with neutrons. For instance, after the end of the bombardment the most abundant ones, like ^13^N (T_1/2_=9.97 min),^128^I (T_1/2_=24.99 min), ^122^I (T_1/2_=3.63 min), ^123^I (T_1/2_=13.2 h) and ^130^I (T_1/2_=12.36 h), decay fast and after 72 h some of them disappear, changing the mutual relative proportions within the product. In effect, the relative ^124^I levels increase from 0.28% to 41.47% and from 0.96 to 43.13% at 10 μA and 15 μA, respectively (Tables 8, 9), as calculated in the Monte Carlo simulations. For the Te enriched target the corresponding increases are from 3.67% to 87.71% and from 8.98% to 87.50% at 10 μA and 15 μA, respectively (Tables 6 and 7). For the natural target the main impurities come from ^126^I, ^125^I and ^130^I (Tables 8 and 9), whereas for the Te enriched target almost exclusively from ^123^I (Tables 6 and 7). According to European Pharmacopeia [31] the contribution from $^{123}$I should be lower or equal to 0.35% of the total activity. We calculated the time when such activity is reached using Monte Carlo simulations. This value was then used to recalculate the activities of the radioisotopes present in the final product vial. The results obtained for the natural and enriched targets are shown in Table 10. As seen from the comparison, enriched tellurium dioxide is more efficient: the post-reaction impurities are at a much lower level (approx. 2%), whereas the main product of the reaction, $^{124}$I, accounts for ca. 98% of the total activity. In the case of natural tellurium dioxide, the impurities are of longer half life times (mainly $^{125}$I with T$_{1/2}$ = 59.4 d and $^{126}$I with T$_{1/2}$ = 12.9 d) than $^{124}$I (T$_{1/2}$ = 4.18 d)—though the final product is more polluted, ^124^I is separated via its distillation from the tellurium oxide matrix.

**Table 10:** The activities for $^{124}I$ and the radionuclidic impurities calculated at the moment when the contribution of the radioactive impurities from $^{123}$I is less than 0.35% of the total activity

Target	Current [μA]	Time from EOB [h]	$^{124}$I [MBq]	$^{124}$I [%]	$^{123}$I [MBq]	$^{123}$I [%]	Impurities [MBq]	Impurities [%]
Natural $^{124}$TeO$_{2}$	10	127	9.62	40.23	0.74	0.35	10.767($^{126}$I), 3.33 ($^{125}$I)	44.98 ($^{126}$I), 13.90 ($^{125}$I)
	15	122	15.318	45.25	0.111	0.35	13.172 ($^{126}$I), 3.737 ($^{125}$I)	38.95 ($^{126}$I), 11.09 ($^{125}$I)
Enriched $^{124}$TeO$_{2}$	10	149	76.627	98.01	0.259	0.35	0.777 ($^{14}$O), 0.259 ($^{123}$I)	0.99 ($^{14}$O), 0.35 ($^{123}$I)
	15	151	117.327	98.34	0.407	0.35	1.184($^{14}$O), 0.407($^{123}$I)	0.98 ($^{14}$O), 0.35 ($^{123}$I)

The separation process adds, however, to the discrepancy between the theoretical and experimental values of the yields. To separate iodine from the TeO_2_ +Al_2_O_3_ target, the sublimation process is employed. This procedure allows more than 75% of the radioactive substance to be extracted from the target material. The distillation efficiency depends on the temperature, the flow rate of the transporting gas, and the operating time. When the radioisotope loss during the separation is included in the calculations, the experimental values are closer to the simulated ones, at least for the proton beam of 10 μA. Our yield results were compared with the literature data and presented in Table 11.

**Table 11:** Comparison of $^{124}$I production yield

Reaction	Target	Energy [MeV]	Yield [MBq/μAh]	References
^124^**Te(p,n)**^124^**I**	**TeO**_2_	**15 → 8**	**32.16 (S)**	[60]
$^{124}$Te(p,n)$^{124}$I	TeO$_{2}$ +Al$_{2}$O$_{3}$ (5%)	11.6 $\rightarrow$ 0	6.88 (E)	[61]
$^{124}$Te(p,n)$^{124}$I	TeO$_{2}$	12.6	13.0 (E)	[62]
$^{124}$Te(p,n)$^{124}$I	TeO$_{2}$ +Al$_{2}$O$_{3}$	13 $\rightarrow$ 9	20 (E)	[19]
$^{124}$Te(p,n)$^{124}$I	TeO$_{2}$ +Al$_{2}$O$_{3}$	12.5 $\rightarrow$ 5	9.0 (E)	[18]
^nat^**Te(p,xn)**^124^**I**	**TeO**_2_	**35 → 22**	**36.63 (S)**	[60]
^nat^**Te(p,xn)**^124^**I**	**TeO**_2_	**10 → 20**	**0.001 (S)**	[63]
$^{nat}$Te(p,xn)$^{124}$I	TeO$_{2}$	29.5 $\rightarrow$ 20	3.95 (E)	[60]
^124^**Te(p,n)**^124^**I**	**TeO**_2_** + Al**_2_O_3_** (5%)**	**14.8**	**14.5 (S)**	*
^nat^**Te(p,xn)**^124^**I**	**TeO**_2_ + **Al**_2_O_3_** (5%)**	**14.8**	**1.58 (S)**	*
$^{nat}$Te(p,xn)$^{124}$I	TeO_2_ + Al_2_O_3_ (5%)	14.8	1.36 (E)	*

*This work, S, simulation; E, experiment

This comparison also favours enriched tellurium dioxide in ^124^I production. However, the natural tellurium dioxide costs ca. 20$ per gram, whereas the tellurium dioxide enriched in $$^{124}$$Te costs ca. 10000$ per gram. In view of such huge difference in the targets’ costs, even the lower activities of ^124^I from the natural Te are not deterrent, the more that they are suitable for PET applications, as the obtained radioactive solution contains $$\beta$$^+^ radioactive isotope. Taking into account the pros and cons of the natural Te vs. enriched Te targets and the range of the possible applications of ^124^I it may be stated that the production methodology of this radionuclide from the natural Te material is worth to be further developed. Even if it gives lower yields, the decision to explore such a route opens the possibility to widen the clinical use of ^124^I and to expand the capability of radioisotope production based on small medical cyclotrons.

## Conclusions

The optimization of radioisotope production is a key issue in maximizing the production yield and minimizing the associated costs. An efficient approach to this problem is the use of Monte Carlo simulations prior to the experiment. It allows to make a cost-free optimization of the most influential production factors, determine the final product contamination, and choose the experimental methodology, especially when the expensive Te enriched targets are planned to be applied in the production. However, a semi-empirical adjustment of the ^124^I production conditions from natural Te is also recommended, especially in view of a vast difference in the targets’ costs. The experimental efficiency for such target revealed to be better than 41% with an average thick target (> 0.8 mm) yield of 1.32 MBq/μAh.

Thus concluding, Monte Carlo is a powerful tool for studying small medical cyclotron radioisotope production performance. Furthermore, this is a cost-efficient approach to studying new radioisotope production mechanisms before investing in costly experimental studies as well as in the case of long-lasting experiments.

## Data Availability

Not applicable.
